# Localizing order to boost signaling

**DOI:** 10.7554/eLife.25375

**Published:** 2017-03-30

**Authors:** Štefan Bálint, Michael L Dustin

**Affiliations:** Kennedy Institute of Rheumatology, University of Oxford, Oxford, United Kingdom; Kennedy Institute of Rheumatology, University of Oxford, Oxford, United Kingdommichael.dustin@kennedy.ox.ac.uk

**Keywords:** super-resolution microscopy, lipid raft, membrane phase separation, Mouse

## Abstract

B-cell receptors form ordered clusters to recruit kinases and exclude phosphatases.

**Related research article** Stone MB, Shelby SA, Núñez MF, Wisser K, Veatch SL. 2017. Protein sorting by lipid phase-like domains supports emergent signaling function in B lymphocyte plasma membranes. *eLife* **6**:e19891. doi: 10.7554/eLife.19891

Biological membranes generally consist of a lipid bilayer that has various receptors and other proteins embedded within it. The lipids in the membrane may be organized into one of two phases: liquid-disordered and liquid-ordered (Simons and Ikonen, 1997). The current view is that most of the membrane is in the liquid-disordered phase; the liquid-ordered regions are small (typically about 20 nanometers across) and span both layers of the membrane (Eggeling et al., 2009; Raghupathy et al., 2015).

Liquid-ordered domains can act as signaling platforms to activate immune cells (Stefanova et al., 1991). In B cells, for example, liquid-ordered domains are recruited to B cell receptors when antigens bind to the receptors: the presence of these domains helps a kinase called Lyn to phosphorylate the receptor, which accelerates the signaling process (Sohn et al., 2006). Now, in eLife, Sarah Veatch and colleagues at the University of Michigan – including Matthew Stone and Sarah Shelby as joint first authors – report how the clustering of B cell receptors (BCRs) creates a membrane domain similar to previously observed liquid-ordered phases, but over a larger area, which helps to activate the receptors (Stone et al., 2017).

Stone et al. used two-color super-resolution microscopy to characterize the lipid environment in the vicinity of the BCR clusters. To visualize the different phases, the ordered and disordered domains were marked with different lipid-linked or transmembrane peptide probes (shortened versions of proteins that are normally found in the cell membrane) linked to a photoactivatable fluorescent protein. Cross-correlation analysis (Sengupta et al., 2011) revealed that liquid-ordered domains are enriched (and hence liquid-disordered domains are depleted) in an area around the clusters. The ordered domains also recruit the Lyn kinase to phosphorylate the receptors, and exclude an enzyme called CD45 (which removes phosphate groups).

The cross-correlation analysis method used by Stone et al. has several advantages over other methods for analyzing images of membranes that contain physiological densities of receptors and low densities of probes (which is necessary to avoid disrupting the lipid phases). For example, it is not susceptible to “over-counting” artifacts related to fluorophore blinking. However, one caveat of this study is that the magnitude of the correlations is quite low, such that the experimental distributions are not far from random patterns. Additional confidence might be gained from coordinate-based co-localization analysis (Malkusch et al., 2012) that provides a nearest neighbor distance, and coclustering methods (Rossy et al., 2014) that provide a co-localization score.

How do the cross-correlation values relate to visual experience? Cross-correlation (Cr) coefficients for B cell antigen receptor with phosphotyrosine (Cr = 8) or with Lyn (Cr = 3) are readily or moderately obvious in images, respectively. In contrast, the Cr values of around 0.8 or 1.2 that are respectively associated with the phase probes labeling the liquid-disordered and liquid-ordered phases near B cell antigen receptor clusters are not readily detected by eye. So the axiom of “seeing is believing” is not applicable.

The cross-correlations can be used to calculate energies needed to account for the non-random organization. The calculated energy is consistent with a moderate restriction on the lateral movement of the receptor through a membrane. Comparing how the peptide phase probes used to mark the liquid-ordered and liquid-disordered phases localize compared with their full length protein counterparts suggests that less than half of the energy involved in recruiting Lyn to B cell antigen receptor clusters is accounted for through effects in the lipid phase. The location of CD45 is influenced by steric clashes with its large extracellular domain (Chang et al., 2016) or the exclusion of its cytoplasmic domain from protein driven phases (Su et al., 2016). However, Stone et al. conclude that all the energy for CD45 exclusion induced by B cell antigen receptor cross-linking comes from membrane phase separation.

Stone et al. present a minimal model for predicting how membrane phase behavior influences B cell signaling. In this model, the clustering of the receptors stabilizes an extended liquid-ordered domain. This domain then recruits Lyn and excludes CD45, leading to the phosphorylation of the receptor (Figure 1A).

**Figure 1.**
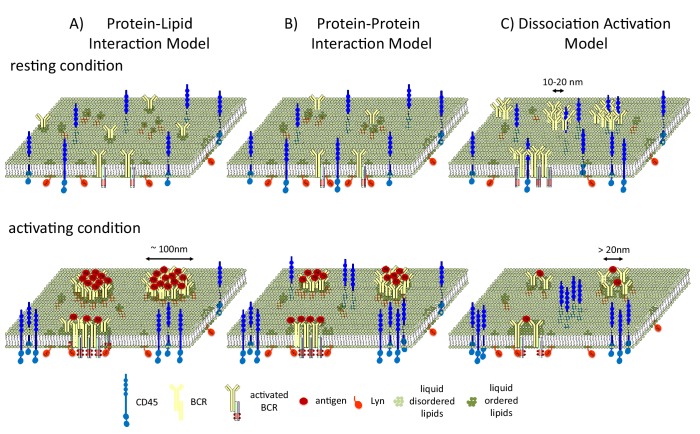
B cell receptors and liquid-ordered domains. (**A**) In the model proposed by Stone et al., B cell receptors (BCRs) cluster and intrinsically interact with liquid-ordered domains. The interaction of the receptors with antigens (Ag) stabilizes an extended liquid-ordered domain (around 100 nanometers in diameter) through protein-lipid interactions that recruit kinases (such as Lyn) and exclude phosphatases (such as CD45). (**B**) In an alternative protein-protein interaction model, B cell receptors do not interact with liquid-ordered domains, but antigen binding causes the receptors to cluster. This initiates interactions between the receptors and lipid-modified kinases, which then recruit liquid-ordered domains that provide further feed forward effects, such as CD45 exclusion, to allow efficient phosphorylation. (**C**) In the dissociation activation model, the receptors cluster to produce a structure in which kinases cannot access the sites they would normally phosphorylate. The binding of antigens to the receptors disrupts the clusters, leading to the recruitment of kinases and the formation of looser clusters that resemble liquid-ordered domains.

Two alternative models have also been used to describe how liquid-ordered domains affect signaling. In the protein-protein interaction model, BCR clustering recruits Lyn, and Lyn then recruits the liquid-ordered domain (Figure 1B). In the dissociation activation model, antigen binding to the receptor disrupts pre-existing clusters (Kläsener et al., 2014). Cluster disruption may also change the lipid environment by allowing more space for the ordered domains (which are around 20 nanometers in diameter) to penetrate into the looser clusters (Figure 1C), which may also be regulated by F-actin mediated restraints (Treanor et al., 2011).

The work of Stone et al. opens up the possibility that lipid phase-like domains may regulate a broad range of signaling pathways. Furthermore, the coupling of localization microscopy and cross-correlation could be extended to investigate other systems – both in the immune system (such as T cell receptors) and beyond. Thus, Stone et al. have entered into a realm of analysis of the seemingly invisible that would give many pause, yet extends the power of localization microscopy to reveal new biology.
